# Centralising specialist cancer surgery services in England: survey of factors that matter to patients and carers and health professionals

**DOI:** 10.1186/s12885-018-4137-8

**Published:** 2018-02-27

**Authors:** Mariya Melnychuk, Cecilia Vindrola-Padros, Michael Aitchison, Caroline S. Clarke, Naomi J. Fulop, Claire Levermore, Satish B. Maddineni, Caroline M. Moore, Muntzer M. Mughal, Catherine Perry, Kathy Pritchard-Jones, Angus I. G. Ramsay, David Shackley, Jonathan Vickers, Stephen Morris

**Affiliations:** 1 Department of Applied Health Research, University College London, Gower Street, London, WC1E 6BT UK; 2 Royal Free London NHS Foundation Trust, Pond Street, London, NW3 2QG UK; 3 Research Department of Primary Care & Population Health, Royal Free Campus, University College London, Rowland Hill St, London, NW3 2PF UK; 4 University College London Hospital NHS Foundation Trust, 47 Wimpole Street, London, W1G 8SE UK; 5 Departments of Urology, Salford Royal NHS Foundation Trust, Stott Lane, Salford, M6 8HD UK; 6 The Christie NHS Foundation Trust, 550 Wilmslow Road, Manchester, M20 4BX UK; 7 Division of Surgical & Interventional Science, University College London, Gower Street, London, WC1E 6BT UK; 8 University College London Hospitals NHS Foundation Trust, 235 Euston Road, London, NW1 2BU UK; 9 Alliance Manchester Business School, University of Manchester, Booth Street East, Manchester, M13 9SS UK; 10 UCLPartners Cancer Programme, 170 Tottenham Court Road, London, W1T 7HA UK; 11 University College London Hospitals Cancer Collaborative, 250 Euston Road, London, NW1 2PG UK; 12 Greater Manchester Cancer, A Block, Christie NHS Foundation Trust, 550 Wilmslow Rd, Manchester, M20 4BX UK; 13 Salford Royal NHS Foundation Trust, Stott Lane, Salford, M6 8HD UK

**Keywords:** Cancer surgery, Implementation, Preferences, Determinants, Service reorganisation, Centralisation, Survey

## Abstract

**Background:**

The centralisation of specialist cancer surgical services across *London Cancer* and *Greater Manchester Cancer*, England, may significantly change how patients experience care. These centres are changing specialist surgical pathways for several cancers including prostate, bladder, kidney, and oesophago-gastric cancers, increasing the specialisation of centres and providing surgery in fewer hospitals. While there are potential benefits related to centralising services, changes of this kind are often controversial. The aim of this study was to identify factors related to the centralisation of specialist surgical services that are important to patients, carers and health care professionals.

**Methods:**

This was a questionnaire-based study involving a convenience sample of patient and public involvement (PPI) and cancer health care professional (HCP) sub-groups in London and Greater Manchester (*n* = 186). Participants were asked to identify which of a list of factors potentially influenced by the centralisation of specialist cancer surgery were important to them and to rank these in order of importance. We ranked and shortlisted the most important factors.

**Results:**

We obtained 52 responses (28% response rate). The factors across both groups rated most important were: highly trained staff; likelihood and severity of complications; waiting time for cancer surgery; and access to staff members from various disciplines with specialised skills in cancer. These factors were also ranked as being important separately by the PPI and HCP sub-groups. There was considerable heterogeneity in the relative ordering of factors within sub-groups and overall.

**Conclusions:**

This study examines and ranks factors important to patients and carers, and health care professionals in order to inform the implementation of centralisation of specialist cancer surgical services. The most important factors were similar in the two stakeholder sub-groups. Planners should consider the impact of reorganising services on these factors, and disseminate this information to patients, the public and health care professionals when deciding whether or not and how to centralise specialist cancer surgical services.

**Electronic supplementary material:**

The online version of this article (10.1186/s12885-018-4137-8) contains supplementary material, which is available to authorized users.

## Background

Since 2011 in London, UK and 2014 in Greater Manchester, UK, cancer care has been provided in integrated cancer systems in which a group of providers come together in a formal way to provide comprehensive cancer care. More recently, these integrated systems are working towards further centralisation of specialist surgical pathways for several cancers, including prostate, bladder, kidney, and oesophago-gastric (OG) cancers, so that specialist complex interventions within these services are provided in fewer hospitals. Implementation of the *London Cancer* centralisations was completed in April 2016, and implementation of the *Greater Manchester Cancer* centralisations is planned for 2017.

Before these most recent changes, potential cancer surgery patients were either referred to their local hospital or the designated specialist service of their cancer network for diagnosis, and either remained there or were referred to a more specialist hospital. The care received by patients and patient volume varied across specialist centres. In the *London Cancer* integrated system (covering a population of 3.2 million in North Central London, North East London and West Essex), patients with prostate and bladder cancer requiring treatment by radical surgery only received robotic surgery in certain specialist centres; the majority of patients requiring surgery for renal cancer underwent surgery in a local non-specialist hospital (performed by a specialist or general urologist), rather than a specialist centre; and patients with OG cancers having major surgery were not guaranteed to see a specialist out of hours or at weekends. These pathways have been reconfigured so that the majority of patients who require them receive specialist surgical services in one specialist hospital in the case of renal, prostate, and bladder cancer and in one of two hospitals in the case of OG cancer.

In the *Greater Manchester Cancer* network (covering a population of 3.1 million in Greater Manchester and East Cheshire), the reconfiguration of specialist surgery is in the design stages and has not yet been implemented. A degree of existing specialisation exists with 3–5 centres offering specialist surgery for each of the described cancer pathways with the plan to move to a model where 1–2 centres provides these in 2018.

Research suggests there is an association between higher volume and better outcomes in many clinical settings [1]. For example, recent research has indicated that centralising acute stroke services into a smaller number of high-volume units is associated with significantly better provision of evidence-based clinical interventions [1], and significantly better clinical outcomes, including patient mortality [2]. Higher volume is associated with better outcomes in specialist surgery for OG cancers [3] and urological cancers [4]. There are longstanding recommendations to implement centralisation of specialist services [5–8], citing potential to increase patient volumes, reduce variations in access, and improve patient outcomes by increasing the likelihood of patients receiving care in hospitals that have a full range of experienced specialists and services to support provision of care.

It has been suggested that among the advantages of a centralised cancer surgery system is the increased patient volume that permits greater specialisation of staff, and greater experience and expertise across teams [9]. Also, under centralised systems specialist services may offer a full range of surgical technologies (e.g. robotic surgery), and equal access to innovative techniques, such as less invasive procedures [10, 11]. Much of the care is still likely to be provided by local hospitals, including diagnosis and ongoing radiotherapy and chemotherapy, with only complex surgery or other interventions (e.g., brachytherapy and cryotherapy) likely to be provided at the specialist centre [12]. Post-centralisation, local hospitals may have closer involvement with specialist centre staff, e.g. joint multi-disciplinary teams (MDTs), and specialists providing training and delivering some outpatient care, with the potential to improve quality of care across the whole system [11, 13]. Possible disadvantages of centralisation are that it is likely to lead to an increase in travel demands on patients and families, and thus may limit people’s access to quality care [14] and to support from family and friends while undergoing specialist surgical treatment.

Little is known about the preferences of patients, the public and health professionals in relation to centralisations of this kind. A review of research evidence indicates that patients are more willing to travel for a number of reasons: to see a specialist; to a hospital with a good reputation; if a condition is serious or urgent; or if the patient is of a higher socioeconomic status. In contrast, older patients and frequent users of services are less willing to travel further, and preferences vary according to the length and inconvenience of the journey [15, 16].

Aligning major system change with stakeholder preferences is likely to increase the likelihood of successful implementation and ongoing sustainability of the changes [17]. This is especially relevant when deciding what changes to implement and how to implement them [17]. Therefore, the aim of this study is to examine the factors that matter to patients and carers and health professionals about the centralisation of specialist surgical services and how these factors vary among different stakeholder groups. The results of this study will subsequently inform a discrete choice experiment that will evaluate how individuals trade off selected attributes of the service [18].

The study is part of a larger project, “Reorganising specialist cancer surgery for the 21^st^ century: a mixed methods evaluation (RESPECT-21)”, that analyses the centralisation of specialised cancer surgical services in the areas covered by *London Cancer* and *Greater Manchester Cancer* [19]. The larger study will address gaps in the evidence about the centralisation of these services, including processes, impact, and cost-effectiveness of changes, as well as identifying lessons that will guide centralisation work in other areas of specialist services (NIHR study reference 14/46/19). The larger study uses a conceptual framework designed to understand the key processes involved in major system change (Additional file 1). These processes include: making the decision to change, developing and agreeing new service models, implementing the changes, and adhering to the new model [19]. Stakeholder preferences, considered in this study, might be especially important during early stages of major system change such as making the decision to change and deciding on which service model to implement.

## Methods

This is a questionnaire-based study of patients, carers and health care professionals.

### Questionnaire design

We identified characteristics of specialist cancer surgical services that might impact on care processes and patient outcomes and could potentially be affected by the centralisation in London and Greater Manchester. We reviewed planning documents covering the development, planning and implementation of the changes to determine characteristics of the care pathway that could vary as a result of the proposed changes and met regularly to create a list of factors that might be affected by the reorganisations [9, 11–13, 20]. Three researchers discussed this list until consensus was reached. This led to the identification of the following 16 factors:

1. Likelihood and severity of complications from surgery that may negatively affect health and increase the length of stay in hospital. Planning documents suggested these might decrease as a result of centralisation if outcomes improve as surgeons and theatre staff in specialist centres carry out more procedures each year [12].
2. Travel time to the hospital where the patient will have surgery or a novel treatment. Centralisation is likely to mean many patients will have to travel further, and therefore for longer, to undergo these procedures; this may also affect the time spent by relatives and friends visiting the hospital [9, 10, 20, 21].
3. Number of specialist cancer surgical procedures carried out per year at the hospital where the patient has surgery; the rationale is evidence showing that patients have better outcomes in centres that carry out a higher number of procedures [9, 10, 20, 21]. Centralisation will reduce the number of centres providing specialist cancer surgery services, and increase the number of procedures carried out per centre.
4. Length of stay in hospital (time from being admitted to hospital for specialist surgery until being discharged). Planning documents suggested length of stay might decrease as a result of fewer complications, more effective specialist team working and familiarity, and the introduction of enhanced recovery programmes [12].
5. Training opportunities for surgical staff. Centres which see larger numbers of patients may be able to provide more training opportunities to staff [12].
6. Having highly trained staff (including surgeons, other doctors and nurses), which may improve outcomes from surgery and reduce the chances of surgical complications. Larger specialist centres may attract highly trained staff, and offer more training opportunities, as above [12, 22].
7. Number of centres where specialist cancer surgery is performed in the local area. As noted, centralisation is designed to reduce the number of centres performing specialist cancer surgery, with the potential advantages and disadvantages described above.
8. Total number of specialised staff providing specialist cancer surgery. Specialist centres will require large teams to monitor patients after surgery, who will have joint appointments or rotate through local hospitals [11].
9. Possible effect on core non-cancer surgical services due to having to share resources and equipment (for example, radiology services). Other non-cancer surgery services may be depleted as resources and equipment were previously shared with specialist cancer surgery services, which will no longer be possible (e.g., loss of specialist surgeons might have an impact on trauma services) [9, 23].
10. Readmissions to hospital after surgery either because the surgery was unsuccessful or because there were complications. As noted, planning documents suggest that centralisation might improve outcomes and reduce readmissions if there are fewer complications after surgery [9, 12].
11. Probability the patient undergoing surgery dies from cancer within the next 12 months. Deaths from cancer might fall if outcomes are better in centres carrying out more operations [9, 10, 18, 19].
12. Having the opportunity to take part in clinical trials of specialist cancer care to test new treatments. High-volume centres are more likely to have the opportunity to participate in clinical trials to evaluate new treatments for cancer [11].
13. Waiting time from referral to surgery, to having the surgical procedure. Centralisation might bring about prompt access to specialists, reducing delays to treatment [12].
14. Having access to staff members from various disciplines (nursing, physiotherapy, dietetics, psychosocial support, radiology, pathology) with specialised skills in cancer surgery to better manage the whole process from start to end. Centralised services are likely to involve specialist multi-disciplinary teams involving input by a range of health professionals into patient care [11].
15. Presence of a core specialist team 24 h a day, 7 days a week, providing on-call emergency care. One of the aims of the reconfigurations is to ensure there is a specialist surgeon available 24/7 to provide care and emergency assistance for complications arising from the surgery.
16. Access to the most up-to-date facilities and medical equipment. Larger specialist centres may be able to attract funding to enhance facilities and equipment [17, 20].

To determine the most important factors that impact preferences for centralisation of specialist cancer services, we designed a questionnaire (see Additional file 2) incorporating the above factors. The text of the questionnaire was reviewed by the Plain English Campaign and three patient representatives of the RESPECT-21 Research Strategy Group to ensure that it was easy to understand.

### Sampling and recruitment

We used a convenience sampling approach, which is commonly used in discrete choice experiments [24, 25], and distributed the questionnaire to several stakeholders groups in London and Greater Manchester. Our sample represented five PPI groups comprising patients (current and former) and carers, and health care professionals from four multi-disciplinary teams involved in managing prostate, bladder, kidney, and OG cancer pathways (who participated in four tumour-specific cancer pathway boards designed to plan, implement, and monitor the reconfiguration of cancer surgical services). The questionnaire was distributed to 86 potential participants either at face-to-face PPI and HCP group meetings following a brief introduction of the study (participants were asked to complete the questionnaire and return it immediately or post it back to the research team) or via email. It was also sent by email to 100 members of a PPI group as part of a newsletter, who were asked to complete it and return it via email. Participants were informed this was a consultation questionnaire about their views on what aspects of the services matter to people most and how important these changes are compared with one another, and that the purpose of this study was to produce a shortlist of the most important items.

### Data collection

Participants were asked to answer two main questions. First, whether any of the 16 factors related to the delivery of cancer surgical services in England were important from their point of view, with possible answers ‘Yes’ or ‘No’ for each of the 16 factors. Second, they were asked to rank the importance of each factor. Participants were also given an option to add items to the original 16. This was important to make sure we did not miss any relevant factors. Participants were asked to identify themselves as patients, carers or health professionals and where they are based (London, Greater Manchester). If participants identified themselves as health care professionals, we asked them to record their specialty.

### Data analysis

We processed all questionnaires and summarised the answers to the questions for all respondents combined and for PPI and HCP groups separately. To summarise responses to the first question, we coded ‘Yes’ responses =1 and ‘No’ responses =0 and summed scores for each factor across all respondents. We divided this sum by the number of respondents who provided a response about that factor as not all respondents valued every factor. We ranked factors according to the mean scores – factors with the highest mean score were ranked more highly. We refer to these results as equal-weighted importance as all factors receiving a ‘Yes’ response are weighted equally in the calculation. For the second question, we summed the ranks for each factor across all participants in each group (rank-weighted importance) and again divided this sum by the number of respondents who provided a response about that factor. As more important factors were allocated a lower rank number (i.e., the most important factor was ranked 1), factors with the lowest mean score were ranked more highly. We presented the ranking of factors according to the equal- and rank-weighed importance for each group and checked for the intersection of the rankings between groups; we preferred the rank-weighted findings as they accounted for the strength of preference of respondents and so focused mainly on these in our description of the results. We measured inter-rater agreement for each ranking using kappa statistics [26].

## Results

We received 52 responses (overall response rate 52/186 = 28%): 19 responses from PPI groups (19/126 = 15%) and 33 responses from HCP groups (33/60 = 55%). Table 1 provides details of the participants’ characteristics categorised by sub-group and location.

**Table 1:** Characteristics of survey participants

	London (*n* = 16)	Greater Manchester (*n* = 36)	Total (*n* = 52)
Sub-group			
PPI	11	8	19
HCP	5	28	33
PPI sub-group			
Patient	10	6	16
Carer	1	1	2
Not answered	0	1	1
HCP sub-group			
Clinical nurse specialist	2	2	4
Dietician	2	0	2
Surgeon	1	5	6
Radiologist	0	2	2
Oncologist	0	5	5
Other	0	4	4
Not answered	0	10	10

*PPI* patient and public involvement, *HCP* health care professional

From the responses received for each group (Additional file 3), we sorted factors by equal-weighted importance (Table 2) and rank-weighted importance (Table 3). Focusing on rank-weighted importance as our preferred measure, on aggregate across all respondents the most important factors affecting preferences for centralisation of specialist cancer surgical services were highly trained staff, likelihood and severity of complications, waiting time for surgery for cancer, and access to staff members from various disciplines with specialised skills in cancer. These were the most important factors for both the PPI and HCP sub-groups, though in a slightly different order. The least important rank-weighted factors overall and in each of the sub-groups were: indirect effect on non-cancer surgical services, number of surgical staff in local area, training opportunities for surgical staff and number of centres in the local area. Results were similar for the equal-weighted ranking with highly trained staff, likelihood and severity of complications, waiting time for surgery for cancer, and access to staff members from various disciplines with specialised skills in cancer all being judged as most important by the HCP sub-group and three of these measures being judged most important by the PPI sub-group (likelihood and severity of complications was ninth most important). At the other end of the ranking, similar factors were also least important, with the exception that for HCP respondents travel time to the hospital was one of the least important attributes and training opportunities for surgical staff were not in the bottom four attributes.

**Table 2:** Equal-weighted ranking of factors (1 = highest ranked, 16 = lowest ranked)

Rank	PPI sub-group (*n* = 19)	HCP sub-group (*n* = 33)	All respondents (*n* = 52)
1	Waiting time for a surgery	Access to staff members from various disciplines with specialised skills in cancer	Waiting time for a surgery
2	Highly trained staff	Highly trained staff	Highly trained staff
3	Access to most up-to-date facilities and equipment	Waiting time for a surgery	Access to staff members from various disciplines with specialised skills in cancer
4	Core specialist team working 24/7	^a^Likelihood and severity of complications	Core specialist team working 24/7
5	^a^Readmissions to hospital	^a^Core specialist team working 24/7	Access to most up-to-date facilities and equipment
6	^a^Access to staff members from various disciplines with specialised skills in cancer	Access to most up-to-date facilities and equipment	^a^Likelihood and severity of complications
7	Number of specialist cancer surgical procedures	^a^Number of specialist cancer surgical procedures	^a^Readmissions to hospital
8	Participation in clinical trials	^a^Readmissions to hospital	Number of specialist cancer surgical procedures
9	Likelihood and severity of complications	Training opportunities for surgical staff	Participation in clinical trials
10	Probability of dying from cancer	Participation in clinical trials	^a^Probability of dying from cancer
11	Travel time to hospital	Length of stay at hospital	^a^Training opportunities for surgical staff
12	^a^Length of stay at hospital	^a^Probability of dying from cancer	Travel time to hospital
13	^a^Number of surgical staff in local area	^a^Indirect effect on non-cancer surgical services	Length of stay at hospital
14	^a^Indirect effect on non-cancer surgical services	Travel time to hospital	Indirect effect on non-cancer surgical services
15	Number of centres in the local area	Number of surgical staff in local area	Number of surgical staff in local area
16	Training opportunities for surgical staff	Number of centres in the local area	Number of centres in the local area
	Kappa = 0.1145	Kappa = 0.1430	Kappa = 0.1295

*PPI* Patient and public involvement, *HCP* Health care professional

^a^denotes tied rankings

**Table 3:** Rank-weighted ranking of factors (1 = highest ranked, 16 = lowest ranked)

Rank	PPI sub-group (*n* = 19)	HCP sub-group (*n* = 33)	All respondents (*n* = 52)
1	**Highly trained staff**	**Highly trained staff**	**Highly trained staff**
2	**Waiting time for a surgery**	**Likelihood and severity of complications**	**Likelihood and severity of complications**
3	^**a**^ **Likelihood and severity of complications**	**Access to staff members from various disciplines with specialised skills in cancer**	**Waiting time for a surgery**
4	^**a**^ **Access to staff members from various disciplines with specialised skills in cancer**	**Waiting time for a surgery**	**Access to staff members from various disciplines with specialised skills in cancer**
5	Core specialist team working 24/7	Number of specialist cancer surgical procedures	Core specialist team working 24/7
6	Access to most up-to-date facilities and equipment	Core specialist team working 24/7	Number of specialist cancer surgical procedures
7	Number of specialist cancer surgical procedures	Readmissions to hospital	Readmissions to hospital
8	Probability of dying from cancer	Probability of dying from cancer	Probability of dying from cancer
9	Readmissions to hospital	Participation in clinical trials	Access to most up-to-date facilities and equipment
10	Travel time to hospital	Access to most up-to-date facilities and equipment	Participation in clinical trials
11	Length of stay at hospital	Travel time to hospital	Travel time to hospital
12	Participation in clinical trials	Length of stay at hospital	Length of stay at hospital
13	*Indirect effect on non-cancer surgical services*	*Number of surgical staff in local area*	*Indirect effect on non-cancer surgical services*
14	*Number of centres in the local area*	^a^ *Training opportunities for surgical staff*	*Number of surgical staff in local area*
15	*Training opportunities for surgical staff*	^a^ *Indirect effect on non-cancer surgical services*	*Training opportunities for surgical staff*
16	*Number of surgical staff in local area*	*Number of centres in the local area*	*Number of centres in the local area*
	Kappa = 0.0114	Kappa = 0.0281	Kappa = 0.0278

*PPI* Patient and public involvement, *HCP* Health care professional

^a^denotes tied rankings. Four most important rank-weighted factors in bold; four least important rank-weighted factors in italics

The kappa statistics overall and for each sub-group were in the range 0.1145 to 0.1430 for the equal-weighted results and 0.0114 to 0.0281 for the rank-weighted results, representing ‘slight’ agreement among rankers in each case [27]. This indicates that, while the top- and bottom-ranked factors overall and in each of the two subgroups were similar, there was considerable heterogeneity regarding the precise ordering of factors within sub-groups and overall.

While the top and bottom four factors were similar for each sub-group, there was more heterogeneity in the ordering between sub-groups with the other factors. The probability of dying from cancer was in this middle group of factors indicating it was less important than the four most important factors described above. Travel time to the hospital was not ranked highly by either sub-group, though as noted this has been raised as a possible negative consequence of centralisation.

Participants were invited to suggest additional factors that were important to them. Three additional factors were suggested, each raised by one participant each (all from the PPI sub-group): availability of free or cheap accommodation for patients and carers; availability of cheap and easy-to-use transport to get to the hospital for patients and carers; and, availability of clear and detailed information for patients and carers about the surgery and follow-up care.

## Discussion

### Summary

This study explored factors influencing preferences for centralisation of specialist cancer surgical services among patients, carers and health care professionals in England. Our results suggest the following factors were ranked as most important:

- Having highly trained staff (including surgeons, other doctors and nurses), which may improve outcomes from surgery and reduce the chances of surgical complications.
- Likelihood and severity of complications from surgery that may negatively affect health and increase the length of stay in hospital.
- Waiting time from referral to surgery to having the surgical procedure.
- Having access to staff members from various disciplines (nursing, physiotherapy, dietetics, psychosocial support, radiology, pathology) with specialised skills in cancer surgery to better manage the whole process from start to end.

We found that different stakeholder groups identified similar factors as being important to them, suggesting that overall patients and carers, and health care professionals, have similar interests and concerns about centralisation. However, while on aggregate similar factors were important to each sub-group, there was considerable within-group and overall heterogeneity in the precise ordering of factors.

As well as identifying factors important to stakeholders when considering centralisation of specialist cancer surgery, this study also identifies factors perceived as being less important by the sample of respondents that might be affected by the reorganisation, including indirect effect on non-cancer surgical services, number of surgical staff in local area, training opportunities for surgical staff and number of centres in the local area. Previous research on the centralisation of cancer services has identified the distance to hospital and travel time as a limiting factor in patients’ decisions to access treatment, especially in the case of patients living in socioeconomically deprived areas [21]. However, the findings of our study seem to support other studies on this topic that found that patients are willing to travel for specialist care [16, 28]. It is important to note that the questionnaire did not ask participants to specifically consider the importance of travel costs, though this will be related to travel time. In addition, travel cost was not raised as a separate issue when participants were given an option to add items to the original list of 16.

### Strengths and limitations

This study has two main strengths. First, the survey questionnaire was rigorously designed, pre-tested, checked for English language problems and is based on a detailed review of planning documents about the centralisations. Second, and to our knowledge, this is the first survey attempting to identify factors related to the centralisation of specialist surgical services that are important to patients, the public and health care professionals.

The main limitations of our study are the small sample size and use of convenience sampling. The survey included only 52 respondents from London and Greater Manchester, and while there were similarities between groups in terms of the ranking of factors, the small sample size and restricted geographical coverage is likely to limit the extent of heterogeneity in responses as well as the generalisability of the findings. Further, the response rate was only 28%. This was mainly driven by the low response rate to the email invitation to participate in the survey (7%); when potential participants were asked to participate face-to-face the response rate was much higher (63%). Another limitation is that we did not collect information on socioeconomic status in the PPI sub-group or age of any of the research participants, to examine how these variables could have influenced the ranking of the factors. We also acknowledge there is likely to be some overlap in the variables included in the analysis (e.g., likelihood and severity of complications and having highly trained staff), though this is not a limitation of the analysis since, while inter-related, the variables capture different attributes of service provision.

### Implications

There are two implications of our study. First, planners who are redesigning services might consider and measure the impact of the reorganisation on the factors identified as being important in this study. Second, they should also disseminate information about these factors to patients, the public and health care professionals when deciding whether or not and how to centralise specialist cancer surgical services. Stakeholder preferences might be especially important during early stages of major system change such as making the decision to change, deciding on which service model to implement, and deciding how to implement the changes (Additional file 1).

### Further research

Further research would be beneficial, repeating this exercise with a larger sample of respondents in different geographical areas, and using face-to-face methods to approach potential participants where possible to maximise responses. Sampling should also include the general public as well as patients and carers and health care professionals, and be undertaken in areas where less centralisation-related activity has taken place. Also, while according to this study there may be agreement between different groups about which are the most important factors affected by centralisation, the relative strength of preference for these factors might vary. Therefore, the results of this study will inform a discrete choice experiment, which will explore strength of preferences for how services are organised for patients, the general public, and healthcare professionals, and how these stakeholders value selected factors.

## Conclusions

This study examines factors important to patients and carers and health professionals in order to inform the implementation of centralisation of specialist surgical services. The most important factors were similar in both stakeholder sub-groups. Planners should consider the impact of reorganising services on these factors, and disseminate information about these factors to patients, to patients, the public and health care professionals when deciding whether or not and how to centralise specialist cancer surgical services.

## Additional files

Additional file 1: Conceptual framework: key components of major system change. Conceptual framework. (DOCX 100 kb)

Additional file 2: Survey questionnaire. Survey questionnaire. (DOCX 38 kb)

Additional file 3: Summary of raw data. Summary of raw data. (DOCX 23 kb)

## Abbreviations

HCP: Health care professionals
MDT: Multi-disciplinary team
OG: Oesophago-gastric
PPI: Patient and public involvement
