# Supplementary material for: Centralising specialist cancer surgery services in England: survey of factors that matter to patients and carers and health professionals
Source: BMC Cancer. 2018 Feb 27;18:226. doi: 10.1186/s12885-018-4137-8 (PMC6389051; doi:10.1186/s12885-018-4137-8)
Supplement: Supplementary file 1 — Conceptual framework: key components of major system change. Conceptual framework. (DOCX 100 kb) [file 12885_2018_4137_MOESM1_ESM.docx]

Additional file 1: Conceptual framework: key components of major system change


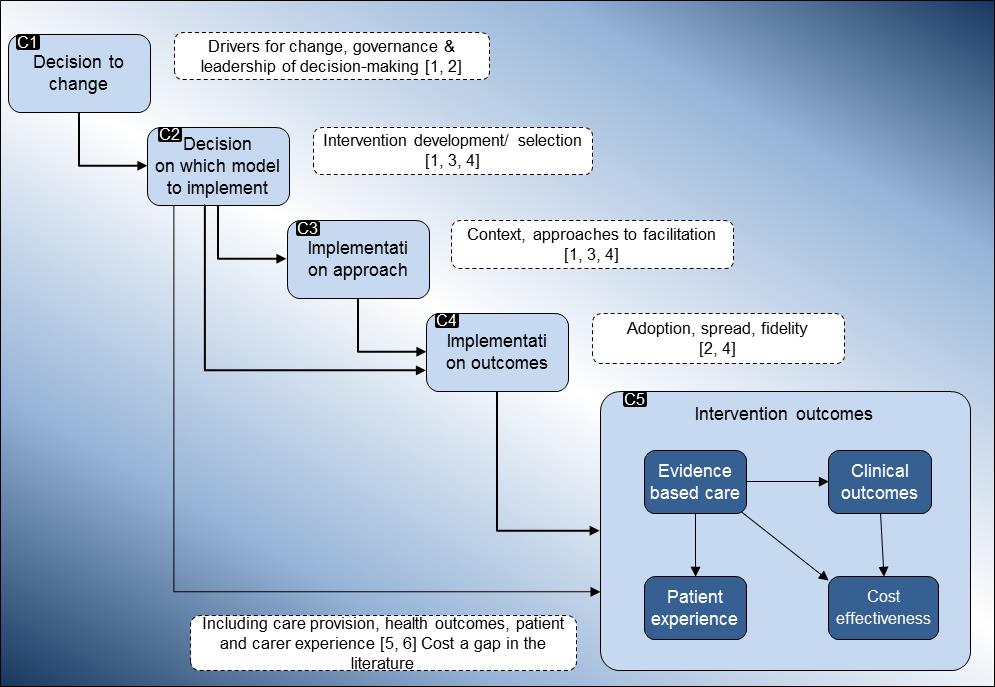


This figure was originally published by Springer in: Fulop NJ, Ramsay AIG, Perry C, Boaden RJ, McKevitt C, Rudd AG, Turner SJ, Tyrrell PJ, Wolfe CDA, Morris S. Explaining outcomes in major system change: a qualitative study of implementing centralised acute stroke services in two large metropolitan regions in England. Implement Sci 2016; 11:80

**References**

1. Kitson AL, Rycroft-Malone J, Harvey G, McCormack B, Seers K, Titchen A. Evaluating the successful implementation of evidence into practice using the PARiHS framework: theoretical and practical challenges. Implement Sci. 2008;3:1.

2. Best A, Greenhalgh T, Lewis S, Saul J, Carroll S, Bitz J. Large-system transformation in health care: a realist review. Milbank Q. 2012;90:421.

3. May C. Towards a general theory of implementation. Implement Sci. 2013;8:18.

4. Tabak RG, Khoong EC, Chambers DA, Brownson RC. Bridging research and practice: models for dissemination and implementation research. Am J Prev Med. 2012;43:337–50.

5. Pronovost P, Goeschel C, Marsteller J, Sexton J, Pham J, Berenholtz S. Framework for patient safety research and improvement. Circulation. 2009;119: 330.

6. Imison C, Sonola L, Honeyman M, Ross S. The reconfiguration of clinical services in the NHS: what is the evidence? London: King's Fund; 2014.
