# Supplementary material for: Centralising specialist cancer surgery services in England: survey of factors that matter to patients and carers and health professionals
Source: BMC Cancer. 2018 Feb 27;18:226. doi: 10.1186/s12885-018-4137-8 (PMC6389051; doi:10.1186/s12885-018-4137-8)
Supplement: Supplementary file 2 — Survey questionnaire. Survey questionnaire. (DOCX 38 kb) [file 12885_2018_4137_MOESM2_ESM.docx]

**Reorganising specialist cancer surgery for the 21^st^ century – a mixed-methods evaluation (RESPECT-21)**

**What are we doing?**

We are working on a study to evaluate the effect of bringing together specialist surgical services for bladder, prostate, kidney and oesophago-gastric cancers across two health-care systems – London Cancer and Manchester Cancer.

The proposed reorganisation of cancer surgery services may change how patients will experience their care, with many having to travel further to receive surgery or specialised investigations, but with a greater choice of treatments and potentially better outcomes. We are carrying out a type of research study called a ‘discrete choice experiment’ to examine what patients, the public, and health professionals think about the changes. We are particularly interested in understanding what changes to services matter to people most, and how important these changes are compared with one another.

**Why do we need your help?**

The study will involve creating a questionnaire and distributing it to patients, the public, and health professionals. First we need to design the questionnaire, and an important step is to draw up a list of changes to services that may be important to people. We have developed a list of possible items and would value your views about whether or not this covers all the important items or if there are any we have missed, and if any of the items on the list are not important and can be dropped. We are aiming to produce a shortlist of five or six of the most important items.

**We would like you to provide some information to help us design our questionnaire.**

**Please click on the box you want to tick.**

**Q1 When thinking about your preferences for reorganising how cancer surgery services are delivered in England, are the following factors important to you? At the bottom of the list, please add any other factors that are important to you that we have missed.**

| **List of factors** | **Yes** | **No** |
| --- | --- | --- |
| 1. Likelihood and severity of complications from surgery that may negatively affect health and increase the length of stay in hospital |  |  |
| 1. Travel time to the hospital where you will have surgery |  |  |
| 1. Number of specialist cancer surgical procedures carried out per year at the hospital where you have surgery |  |  |
| 1. Length of stay in hospital (time from being admitted to hospital for surgery until being discharged) |  |  |
| 1. Training opportunities for surgical staff |  |  |
| 1. Having highly trained staff (including surgeons, other doctors and nurses), which may improve outcomes from surgery and reduce the chances of surgical complications |  |  |
| 1. Number of centres where specialist cancer surgery is performed in the local area |  |  |
| 1. Total number of staff providing specialist cancer surgery in the local area |  |  |
| 1. Possible effect on core surgical services not delivering the cancer surgery due to having to share resources and equipment (for example, radiography) |  |  |
| 1. Readmissions to hospital after surgery either because the surgery was unsuccessful or because there were complications |  |  |
| 1. Probability patient undergoing surgery dies from cancer within the next 12 months |  |  |
| 1. Having the opportunity to take part in clinical trials of specialist cancer care to test new treatments |  |  |
| 1. Waiting time from referral to surgery to having the surgical procedure |  |  |
| 1. Having access to staff members from various disciplines (nursing, physiotherapy, dietetics, psychosocial support, radiology, pathology) with specialised skills in cancer surgery to better manage the whole process from start to end |  |  |
| 1. Presence of a core specialist team so someone is available 24 hours a day, seven days a week and providing on-call emergency care |  |  |
| 1. Access to the most up-to-date facilities and medical equipment |  |  |
|  |  |  |
|  |  |  |
|  |  |  |

**Q2 Please rank the level of importance (1=most important, 2=second most important, and so on) of the following factors when thinking about your preferences for reorganising how cancer surgery services are delivered in England.**

| **List of factors** | **Ranking** |
| --- | --- |
| 1. Likelihood and severity of complications from surgery that may negatively affect health and increase the length of stay in hospital |  |
| 1. Travel time to the hospital where you will have surgery |  |
| 1. Number of specialist cancer surgical procedures carried out per year at the hospital where you have surgery |  |
| 1. Length of stay in hospital (time from being admitted to hospital for surgery until being discharged) |  |
| 1. Training opportunities for surgical staff |  |
| 1. Having highly trained staff (including surgeons, other doctors and nurses), which may improve outcomes from surgery and reduce the chances of surgical complications |  |
| 1. Number of centres where specialist cancer surgery is performed in the local area |  |
| 1. Total number of staff providing specialist cancer surgery in the local area |  |
| 1. Possible effect on core surgical services not delivering the cancer surgery due to having to share resources and equipment (for example, radiography) |  |
| 1. Probability patient undergoing surgery dies from cancer within the next 12 months |  |
| 1. Readmissions to hospital after surgery either because the surgery was unsuccessful or because there were complications |  |
| 1. Having the opportunity to take part in clinical trials of specialist cancer care to test new treatments |  |
| 1. Waiting time from referral to surgery to having the surgical procedure |  |
| 1. Having access to staff members from various disciplines (nursing, physiotherapy, dietetics, psychosocial support, radiology, pathology) with specialised skills in cancer surgery to better manage the whole process from start to end |  |
| 1. Presence of a core specialist team so someone is available 24 hours a day, seven days a week and providing on-call emergency care |  |
| 1. Access to the most up-to-date facilities and medical equipment |  |
|  |  |
|  |  |
|  |  |

**Q3 Tell us about you (Tick as appropriate.)**

1. **I am a:**

patient carer health-care professional

Other: ______________________________________

**B) If you are a patient, are you:**

a cancer patient? another type of patient? _____________________________

1. **If you are a carer, are you caring for:**

a cancer patient? another type of patient? _____________________________

1. **If you are a health-care professional, please tell us your specialty:** ________________________________________________________________________
2. **I am based in:**

London Greater Manchester Other: ___________________

**F) Date this feedback form was completed:** ___/___/___

DD/MM/YY

**Q4 Do you have any other thoughts or comments on the questions above or the project in general?**

|  |
| --- |
|  |
|  |

**Thank you for taking the time to fill in this feedback form.**

**Please return it to Mariya Melnychuk:** [m.melnychuk@ucl.ac.uk](mailto:m.melnychuk@ucl.ac.uk)
