# Supplementary material for: Centralising specialist cancer surgery services in England: survey of factors that matter to patients and carers and health professionals
Source: BMC Cancer. 2018 Feb 27;18:226. doi: 10.1186/s12885-018-4137-8 (PMC6389051; doi:10.1186/s12885-018-4137-8)
Supplement: Supplementary file 3 — Summary of raw data. Summary of raw data. (DOCX 23 kb) [file 12885_2018_4137_MOESM3_ESM.docx]

**Table S1. Equal-weighted importance of factors, PPI group**

| **Factor** | **Mean score** | **n** |
| --- | --- | --- |
| Waiting time for a surgery | 1.000 | 19 |
| Highly trained staff | 0.947 | 19 |
| Access to most up-to-date facilities and equipment | 0.947 | 19 |
| Core specialist team working 24/7 | 0.895 | 19 |
| Readmissions to hospital | 0.842 | 19 |
| Access to staff members from various disciplines with specialised skills in cancer | 0.842 | 19 |
| Number of specialist cancer surgical procedures | 0.789 | 19 |
| Participation in clinical trials | 0.778 | 18 |
| Likelihood and severity of complications | 0.737 | 19 |
| Probability of dying from cancer | 0.684 | 19 |
| Travel time to hospital | 0.632 | 19 |
| Length of stay at hospital | 0.526 | 19 |
| Number of surgical staff in local area | 0.526 | 19 |
| Indirect effect on non-cancer surgical services | 0.526 | 19 |
| Number of centres in the local area | 0.474 | 19 |
| Training opportunities for surgical staff | 0.421 | 19 |

**Table S2. Equal-weighted importance of factors, HCP group**

| **Factor** | **Mean score** | **n** |
| --- | --- | --- |
| Access to staff members from various disciplines with specialised skills in cancer | 1.000 | 33 |
| Highly trained staff | 0.970 | 33 |
| Waiting time for a surgery | 0.969 | 32 |
| Likelihood and severity of complications | 0.939 | 33 |
| Core specialist team working 24/7 | 0.939 | 33 |
| Access to most up-to-date facilities and equipment | 0.906 | 32 |
| Number of specialist cancer surgical procedures | 0.879 | 33 |
| Readmissions to hospital | 0.879 | 33 |
| Training opportunities for surgical staff | 0.818 | 33 |
| Participation in clinical trials | 0.818 | 33 |
| Length of stay at hospital | 0.697 | 33 |
| Probability of dying from cancer | 0.667 | 33 |
| Indirect effect on non-cancer surgical services | 0.667 | 33 |
| Travel time to hospital | 0.636 | 33 |
| Number of surgical staff in local area | 0.545 | 33 |
| Number of centres in the local area | 0.394 | 33 |

**Table S3. Equal-weighted importance of factors, all respondents**

| **Factor** | **Mean score** | **n** |
| --- | --- | --- |
| Waiting time for a surgery | 0.980 | 51 |
| Highly trained staff | 0.962 | 52 |
| Access to staff members from various disciplines with specialised skills in cancer | 0.942 | 52 |
| Core specialist team working 24/7 | 0.923 | 52 |
| Access to most up-to-date facilities and equipment | 0.922 | 51 |
| Likelihood and severity of complications | 0.865 | 52 |
| Readmissions to hospital | 0.865 | 52 |
| Number of specialist cancer surgical procedures | 0.846 | 52 |
| Participation in clinical trials | 0.804 | 51 |
| Probability of dying from cancer | 0.673 | 52 |
| Training opportunities for surgical staff | 0.673 | 52 |
| Travel time to hospital | 0.635 | 52 |
| Length of stay at hospital | 0.635 | 52 |
| Indirect effect on non-cancer surgical services | 0.615 | 52 |
| Number of surgical staff in local area | 0.538 | 52 |
| Number of centres in the local area | 0.423 | 52 |

**Table S4. Rank-weighted importance of factors, PPI group**

| **Factor** | **Mean score** | **n** |
| --- | --- | --- |
| Highly trained staff | 3.750 | 16 |
| Waiting time for a surgery | 4.563 | 16 |
| Likelihood and severity of complications | 5.333 | 15 |
| Access to staff members from various disciplines with specialised skills in cancer | 5.533 | 15 |
| Core specialist team working 24/7 | 6.000 | 15 |
| Access to most up-to-date facilities and equipment | 7.286 | 14 |
| Number of specialist cancer surgical procedures | 7.688 | 16 |
| Probability of dying from cancer | 7.769 | 13 |
| Readmissions to hospital | 7.938 | 16 |
| Travel time to hospital | 8.857 | 14 |
| Length of stay at hospital | 9.333 | 15 |
| Participation in clinical trials | 9.643 | 14 |
| Indirect effect on non-cancer surgical services | 10.667 | 12 |
| Number of centres in the local area | 10.929 | 14 |
| Training opportunities for surgical staff | 11.429 | 14 |
| Number of surgical staff in local area | 11.692 | 13 |

**Table S5. Rank-weighted importance of factors, HCP group**

| **Factor** | **Mean score** | **n** |
| --- | --- | --- |
| Highly trained staff | 2.636 | 33 |
| Likelihood and severity of complications | 4.750 | 32 |
| Access to staff members from various disciplines with specialised skills in cancer | 4.939 | 33 |
| Waiting time for a surgery | 5.276 | 29 |
| Number of specialist cancer surgical procedures | 6.152 | 33 |
| Core specialist team working 24/7 | 6.250 | 32 |
| Readmissions to hospital | 7.281 | 32 |
| Probability of dying from cancer | 8.143 | 28 |
| Participation in clinical trials | 8.290 | 31 |
| Access to most up-to-date facilities and equipment | 8.500 | 32 |
| Travel time to hospital | 9.290 | 31 |
| Length of stay at hospital | 9.375 | 32 |
| Number of surgical staff in local area | 9.867 | 30 |
| Training opportunities for surgical staff | 10.161 | 31 |
| Indirect effect on non-cancer surgical services | 10.161 | 31 |
| Number of centres in the local area | 10.839 | 31 |

**Table S6. Rank-weighted importance of factors, all respondents**

| **Factor** | **Mean score** | **n** |
| --- | --- | --- |
| Highly trained staff | 3.000 | 49 |
| Likelihood and severity of complications | 4.936 | 47 |
| Waiting time for a surgery | 5.022 | 45 |
| Access to staff members from various disciplines with specialised skills in cancer | 5.125 | 48 |
| Core specialist team working 24/7 | 6.170 | 47 |
| Number of specialist cancer surgical procedures | 6.653 | 49 |
| Readmissions to hospital | 7.500 | 48 |
| Probability of dying from cancer | 8.024 | 41 |
| Access to most up-to-date facilities and equipment | 8.130 | 46 |
| Participation in clinical trials | 8.711 | 45 |
| Travel time to hospital | 9.156 | 45 |
| Length of stay at hospital | 9.362 | 47 |
| Indirect effect on non-cancer surgical services | 10.302 | 43 |
| Number of surgical staff in local area | 10.419 | 43 |
| Training opportunities for surgical staff | 10.556 | 45 |
| Number of centres in the local area | 10.867 | 45 |
